# Sialolithiasis. Proposal for a new minimally invasive procedure: Piezoelectric surgery

**DOI:** 10.4317/jced.51253

**Published:** 2014-07-01

**Authors:** Victoria Pastor-Ramos, Alfonso Cuervo-Díaz, Luis Aracil-Kessler

**Affiliations:** 1 MD, DDs, PhD. Private practice, Madrid. Spain; 2 DDS. Private practice. Dental clinic Menedent, Madrid. Spain; 3 MD, DDs, PhD. Associate Professor. Department of Stomatology, School of Dentistry, The Complutense University of Madrid. Spain

## Abstract

Sialolithiasis is the presence of stones in the ducts of the salivary glands. Most episodes are unique, and 60-80% are located exclusively in the main excretory duct. The main clinical manifestations are swelling and pain typically before, during or after meals that decreases if the obstruction is not complete. The highest prevalence of lithiasis is in the submandibular gland -87%-, whose secretion is more viscous, followed by the parotid gland -10%- and finally the sublingual gland -3%-. The most significant consequences are caused by the prolonged blockage of the duct by a stone, which can produce a persistent ductal dilatation with a swelling that does not subside, and could lead to the complete degeneration of the parenchyma, becoming a hot spot where secondary infections may occur, leading to acute bacterial sialadenitis or glandular abscesses. Treatment options range from a single probing extraction, extraction with sialographic control using the sialoendoscope, LASER intraductal lithotripsy, lithotripsy extracorporeal shock wave (ESWL), to the surgical techniques combining open duct with endoscopic or glandular removal. We propose, with regard to a case, the use of a simple piezoelectric device which, tunnelling through the glandular channel by the ostium, allows stone fragmentation, without damaging the surrounding soft tissue. Stone removal by this less invasive method reduces the need for more complex and expensive techniques. The postoperative course without retraction of the ostium, and the regaining of functionality is favourable.

** Key words:**Calculus, lithotripsy, minimally invasive therapy, piezoelectric surgery, salivary glands, soft tissues.

## Introduction

Sialolithiasis is the presence of stones in the major or minor salivary glands. Most of calculi are located in the extraglandular paths of the ducts at a ratio of 7:3. The estimated rate of occurrence is 1:10.000 to 1:20 000, and the man: woman ratio is 2:1. Microliths may be expelled or continue to grow until the duct is obstructed. Usually the stone is an isolated event, with little recurrence. Histopathological examination of extracted glands shows 50% with a normal pattern (1).

Simple radiography can confirm up to 80% of cases, the remaining 20% may need ultrasound, computed tomography [CT], sialography, sialography combined with computed axial tomografhy [sialoTAC], magnetic resonance imaging [MRI] or sialoendoscopy.

Sialolithiasis treatment involves the removal of the calculus, which restores total function in 80% of cases, even with acute inflammation. Only if the blockage is in a very deep zone or if there is general sickness, should treatment be delayed. Conservative treatment consists of abundant hydration, the administration of sialogogues -lemon juice, ascorbic acid-, non-steroidal anti-inflammatory drugs [NSAIDs], local heat and manual stimulation. When ineffective, it is necessary to use interventional procedures which have improved in recent years, by the incorporation of minimally invasive techniques (2).

A review of literature shows as treatment options:

1.- Gentle probing through the ostium of the duct with a blunt instrument, it may be sufficient to remove the

stone.

2.- Interventional sialography. Already described in 1990, this is the blind extraction of a calculus via the can

liculus with Dormia baskets and sialographic control.

3.- Therapeutic sialendoscopy: Performed for the first time in the early nineties (3). This is carried out with small

endoscopes -1.2-1.3 mm diameter-. Once the stone is accessed, it is collected with the clamp and removed or

fragmented. It is the procedure of choice for calculi of small and medium size, with a high success rate -up to

75%- with glandular preservation (4). However, 30% of patients will require other approaches (5) espe-cially if

stones are large or deeply located, and cannot be fragmented. The sialendoscope handling is delicate and requires

experience. The risks of this technique are perforation and vascular or neural damage. The progression in the

channel should be performed only in appropriate viewing conditions. Iatrogenic perforation can lead to wid

spread inflammation of the base of the mouth, with potential life threatening implications (6).

4.- Shock wave therapy ESWL [extra-corporeal shock-wave lithotripsy]. Described in 1986 for salivary glands

(7) and clinically developed in 1989 (8) .The objective of this technique is the fragmentation of the stone into pieces no greater than 1.2 mm in order to eliminate it. This system uses ultrasonic waves to break up stones, via a small emitter suitable for salivary glands. It is intended for moderately large or intraparenchymal stones. Usually it is necessary to have up to three sessions to complete the treatment. The complete success as the primary treatment is 40% in submandibular cases and 70% in parotid cases (9,10)

5.- Intraductal lithotripsy. Already in 1990 (3) LASER beams were being used to fragment stones through the endoscope in submaxillary lithiasis. In this case the shock waves reach the calculus through a tube in the duct guided by the sialoendoscope.

The most commonly used LASER are the holmium YAG LASER, which has an increased risk of soft tissue damage by thermal effect causing perforations or fistulas, and the Pulsed Dye LASER which is less risky, but very expensive.

6.- Surgery: despite the techniques described above, up to 10% of parotid lithiasis cannot be sucessfully treated, and it is necessary to perform surgery. If the stone is close to the ostium, the dissection and the duct opening may be therapeutic. The narrowing of the duct can be a result of this type of surgery within the duct. Papillotomy should be avoided in the case of the Stenon duct (11). In deeper located calculus it may be removed just the superficial lobe, external to the division of the branches of the facial nerve -superficial parotidectomy- or the superficial and deep lobe, preserving the facial nerve -total parotidectomy-. Complications of parotid surgery, even superficial ones, are common, and may include temporary or permanent facial palsy, Frey syndrome -periprandial skin sweating-, anaesthesia of the ear, salivary fistulas or unsightly scars.

7.- Our proposal: Piezoelectric Surgery. Developed by Vercellotti (12) in 2001 as a new technique to cut bone in sinus lifts without damaging the membrane of Schneider . With the piezoelectric scalpel we can fragment the stone, preserving the soft tissue of the parotid duct.

## Case Reports

**Figure 1:**
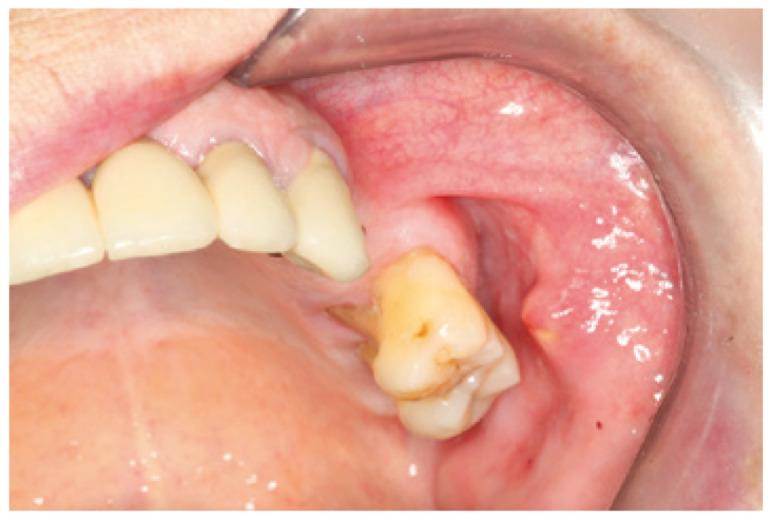
Calculus in the mouth at the start.

**Figure 2:**
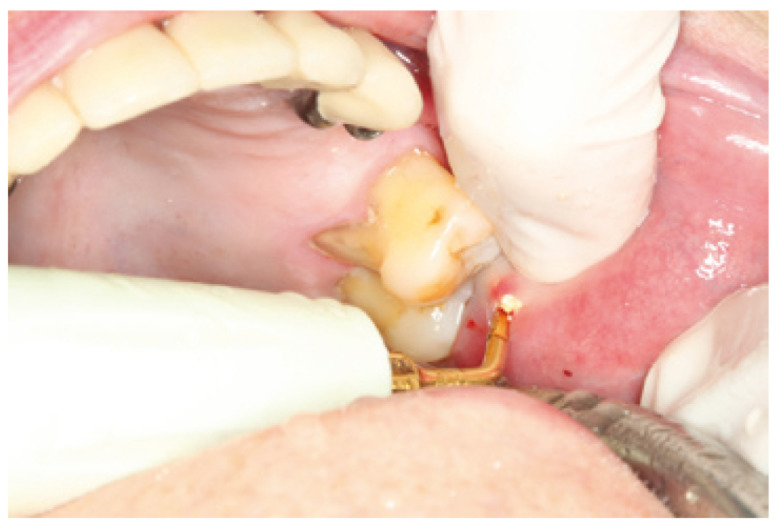
Stone fragments during removal.

**Figure 3:**
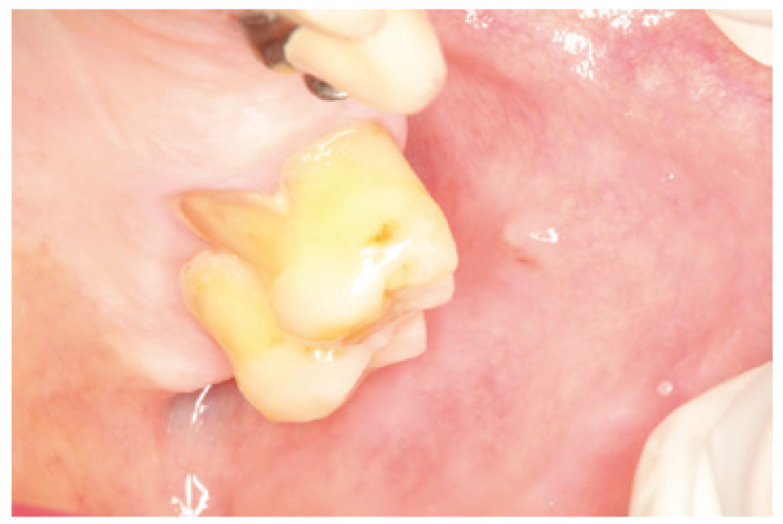
Appearance of the Stenon two months after surgery. The function is normal.

We report the case of a 92 year old female patient, with good overall health, who had experienced an isolated episode of non inflammatory increase volume of the parotid gland 2 months earlier. We suspected a parotid lithiasis that had evolved over a period of several months. At the time of examination, a hard consistency formation with 9 mm lenght and an irregular surface, was found protruding near the emergence of the left parotid duct (Fig. 1), which could not be removed by squeezing. Plain radiographs showed a radiopaque mass located in the thickness of the excretory duct area. The left parotid gland had a normal size, and drained clear saliva. The accessibility to the stone by the ostium, and the predictable simplicity and low invasiveness of the procedure, suggested trying the fragmentation with the piezoelectric scalpel. This was performed under local anaesthesia with lidocaine, tunnelling the Stenon (Fig. 2) by inserting a short angled device in the piezoelectric scalpel normally used for sinus lift. The result was the fragmentation of the stone into small elements which could then be removed manually. On follow up examination 6 months later, the patient remains asymptomatic, there are no calculus in the Stenon, she drains clear saliva and both mucosa and papilla are a normal colour (Fig. 3).

## Discussion

The most significant consequences of sialolithiasis are caused by the prolonged blockage of the duct by a stone, which can produce a persistent ductal dilatation with a swelling that does not subside, and could lead to the complete degeneration of the parenchyma gland. If the glands are no longer functional they become a hot spot where secondary infections may occur, leading to acute bacterial sialadenitis or glandular abscesses which cause severe pain, fever and discomfort. Evidence suggests that after removal of a stone the function of the gland recovers (13).

The traditional approach to obstructive salivary disorders was duct dilatation, incision and dissection in the case of distal stones -sometimes followed by marsupialisation, with the risk of postoperative stenosis- (14) but if possible, papillotomy should be avoided in the case of the Stenon duct.

New minimally invasive techniques have been developed, helping the physician to perform a gland-preserving form of therapy. The results of which indicate that only about 3% of patients subsequently require excision (9). In the case of big stones, it is necessary to fragment them before extraction, which can be done with ESWL, -less useful in big ones-, intraductal lithotripsy with LASER –complex and expensive- or sialendoscopy –which fragment them mechanically, also less useful in big ones-.

To ensure a safe technique, but also to resolve a case of a big stone in the distal portion of the parotid duct, ultrasound piezosurgery can be used. This is a minimally invasive technique that decreases the damage of surrounding soft tissues.

The piezoelectric effect was described by brothers Pierre and Jacques Curie in 1880, describing how certain natural crystals generate an electric potential in response to mechanical stress. A year later, Lippmann discovered the inverse piezoelectric effect. This explains how certain materials, when subjected to an electrical field, can deform according to the polarity and intensity of the same, and generate a small ultrasonic pulse. Surgical piezoelectric instruments operate on this inverse principle.

This surgical technique has progressed most in the last 10 years with the studies of Vercellotti (14). The main advantages of piezoelectric surgery applied to hard tissue are to protect the soft tissue [nerves, vessels, mucous], excellent visibility of the surgical field, and minimal bleeding, together with low vibration and noise. Its applications in oral surgery, include endodontic surgery, periodontal surgery, complex tooth extraction, implants, bone grafts extractions, alveolar nerve transposition, removal of cysts, orthodontic-surgical procedures, sinus lift (14), distraction osteogenesis, ridge expansion, etc. It is also useful in orthognathic surgery, craniofacial, spinal, orthopedic, orbital (15), hand surgery, etc.

Frequencies of 25 to 29 kHz cut only mineralized tissue, and soft tissues are cut at frequencies higher than 50 kHz (14). Vibrating the tip in a range of 60 to 200 microns, allows a clean cut in hard tissue, without blood vessels breakage (15) or causing overheating which damages cell vitality. It could damage delicate structures only if high intensity direct mechanical pressure is exerted, or if irrigation is not sufficient.

The restriction of this technique is its dependence on the accessibility of the stone, because the ones located deeper in the gland will not be accessible to the tip of the device. The device used is a VarioSurg Piezo Bone Surgery NSK. It consists of a piezoelectric hand-piece with an adjustable irrigation system connected to the main unit. The selection of power and frequency modulation is done from the control panel. The inserts coated with TiN [titanium nitride] improve cutting power while minimizing heat production. In “boosted” mode it produces a digital modulation of the oscillation of alternating higher frequencies with pauses, which prevents collision with the bone and prevents overheating while maintaining optimum cutting ability.

The application of this technique for the fragmentation of stones accessible via the ostium, is attractive for its good safety record in preserving the surrounding delicate soft tissue. In this case, the technique has been performed blind, but it might be possible to combine it with sialoendoscopy for fragmentation of deeper large stones with less complexity than the YAG LASER, and a lower cost than Pulsed Dye LASER.

For the patient, it is a simple procedure that allows treatment in a single session, with subsequent radiographic control. In this case, it has evolved well without side effects or follow up treatment needed, allowing greater patient satisfaction. It is a simple technique that requires an affordable learning curve for the operator. The iatrogenic possibilities are limited, and in any case, have a lesser impact than in open technique procedures. It will be necessary to see in future studies the applicability in case series to designate indications and potential risks.
